# Why Are Some Plant Genera More Invasive Than Others?

**DOI:** 10.1371/journal.pone.0018654

**Published:** 2011-04-11

**Authors:** John Paul Schmidt, John M. Drake

**Affiliations:** Odum School of Ecology, University of Georgia, Athens, Georgia, United States of America; University of Zurich, Switzerland

## Abstract

Determining how biological traits are related to the ability of groups of organisms to become economically damaging when established outside of their native ranges is a major goal of population biology, and important in the management of invasive species. Little is known about why some taxonomic groups are more likely to become pests than others among plants. We investigated traits that discriminate vascular plant genera, a level of taxonomic generality at which risk assessment and screening could be more effectively performed, according to the proportion of naturalized species which are pests. We focused on the United States and Canada, and, because our purpose is ultimately regulatory, considered species classified as weeds or noxious. Using contingency tables, we identified 11 genera of vascular plants that are disproportionately represented by invasive species. Results from boosted regression tree analyses show that these categories reflect biological differences. In summary, approximately 25% of variation in genus proportions of weeds or noxious species was explained by biological covariates. Key explanatory traits included genus means for wetland habitat affinity, chromosome number, and seed mass.

## Introduction

Invasion by non-indigenous plants is a dominant driver of ecosystem change and a major problem for environmental management worldwide [1], [2], [3]. Many species invasions result from introductions associated with human industry and development [4], [5], [6]. Therefore, determining patterns in the kinds of non-native plants that establish and spread is an important step toward improved prediction, surveillance and management. Some of these patterns are taxonomic, suggesting that “invasiveness syndromes” might be evolutionarily stable. For instance, a comparative review of 19 studies [7] reports that non-random taxonomic patterns at the level of family are common. Similar progress has been made toward identifying traits linked to invasiveness among plant species [7], [8], [9], [10], [11], [12], although many authors consider these advances to lack the level of generalization needed for prediction of pest species in a regulatory context [10], [13]. Such studies typically find the success of plant invaders to be related to short life cycle, abiotic (mostly wind) dispersal, large native range size, clonality, occupation of disturbed habitats, and time since introduction [7]. These results are complemented by studies that collectively find strong support for the role of biotic traits such as height, vigorous vegetative growth, early and extended flowering, and attractiveness to humans [14]. Thus, a relatively detailed picture of the traits that confer invasiveness at a species level is beginning to emerge. While recent work suggests [15], [16] that genus-level attributes can contribute to success in naturalization and subsequent abundance, knowledge of trait patterns is lacking at this level, where biological specificity remains considerable and the scope for regulatory generalization is maximal.

Here we report results of a study of taxonomic patterns of plants designated economic pests at the genus level. Noting that most species identified as pests in the U.S. and Canada by the Plants National Database (http://plants.usda.gov/, maintained by the USDA Natural Resources Conservation Service) occur in only 500 of 1638 vascular plant genera, we first investigated over-representation of pest species in these genera. Then, having identified genera with a propensity for invasiveness, we asked to what degree invasion success as a property of a genus can be related to biotic traits, aggregating over 3,794 species in 760 genera (173 families, 50 orders) represented by more than one naturalized species. While a useful taxonomic level at which to implement regulatory controls, attributes of genera which may confer a propensity to produce pests once introduced into new regions has been little investigated. To identify relationships between dependent variables and explanatory variables where patterns were expected to be complex and involve interactions and trade-offs, we investigated different machine learning approaches.

Ultimately, the evidence that invasiveness – here defined as propensity to naturalize and do economic damage - is mechanistically linked to biological traits will come when ecological knowledge is encapsulated in informational technologies (e.g., models, algorithms, and decision procedures) that accurately predict what species will become invasive when introduced to a region [9], [17]. The aim of our study was to relate categories that reflect the degree of economic and environmental damage (weeds vs. species legally recognized as pests) to biotic traits, and phylogenetic relatedness, of the species. Aiming ultimately to assist regulators, our strategy has been to establish which traits are related to “weediness” as a general category of species that are economically damaging, and which traits as associated with more serious pests that are legally defined as noxious. In this way, we investigate to what degree the economic categories “weed” and “noxious” correspond to ecological and taxonomic groupings.

## Results and Discussion

### Patterns of invasiveness among genera and at higher taxonomic levels

Weeds and noxious species are over-represented within genera of angiosperms (Table 1). Two genera, *Cuscuta* and *Melastoma*, were disproportionate in number of weeds while 4 genera, *Carduus*, *Dipsacus*, *Miscanthus*, *Onopordum*, *Solanum*, were disproportionate in noxious species, and 5 genera, *Carduus*, *Centaurea*, *Prosopsis*, *Salsola*, and *Sorghum*, were disproportionate in both weeds and noxious species. While not statistically significant due to multiple comparisons, 40 additional genera were disproportionately weedy and 27 genera disproportionately noxious at the α = 0.05 level for individual comparisons (Appendix S1). We believe this to be the first study to document genus-level taxonomic patterns of invasiveness across such a broad group of invasive plant species. Of the 11 invasive genera, 4 were C_4_, and 3 of these were C_4_ perennial grasses. Three of the 11 genera were composites, members of the second largest family of flowering plants in terms of species. Finally, four of the 11 genera contained biennials, while only 2 genera were primarily annual. Biennials are, therefore, particularly over-represented given that they are only present in 131 of 1,638 (8%) introduced genera. Most of the invasive genera are primarily pests of the arid and semi-arid rangelands of the U.S. West (*Cuscuta*, a parasitic genus, and *Melastoma* are exceptions). Thus, we found taxonomic patterns at the genus-level and higher for vascular plant species listed as pests with these patterns applying, particularly for genera, to pests of grasslands, savannas, and rangelands.

**Table 1:** Results of contingency table analysis.

genus	level	# intros	# Weed	# State	life-form	traits	family	order
*Carduus* *	Noxious, Weed	6	5	5	forb	mosty biennial, or perennial, 2 annual, wind-dispersed	Asteraceae	Asterales
*Centaurea*	Noxious, Weed	30	12	11	forb	annual, biennial, perennial	Asteraceae	Asterales
*Cuscuta* *	Weed	6	6	6	vines	parasitic, vine	Cuscutaceae	Solonales
*Dipsacus*	Noxious	3	3	3	forb	biennial	Dipsacaceae	Dipsacales
*Melastoma*	Weed	3	3	1	shrubs, trees	vertebrate dispersed, 1 facultative wetland	Melastomataceae	Myrtales
*Miscanthus*	Noxious	3	3	3	grass	C_4_, perennial, wind-dispersed, facultative wetland	Poaceae	Cyperales
*Onopordum*	Noxious	3	3	3	forb	biennial, wind-dispersed	Asteraceae	Asterales
*Prosopis*	Noxious, Weed	7	6	6	shrubs, trees	N-fixer, 2 facultative wetland	Fabaceae	Fabales
*Salsola*	Noxious, Weed	6	5	5	forb	C_4_, annual, 1 facultative wetland	Chenopodiaceae	Caryophyllales
*Solanum*	Noxious	34	11	3	forbs, shrubs, trees, vines	mostly perennial, some annuals	Solonaceae	Solonales
*Sorghum*	Noxious, Weed	3	3	3	grass	C_4_, perennial, 2 facultative wetland	Poaceae	Cyperales

*Genus is listed rather than individual species.

Genera listed are disproportionately high in the number of species listed as weeds, or state- or federal noxious species per number of species introduced.

We included measures of phylogenetic relatedness along with biotic traits in boosted regression tree analyses to test whether combining both sets of explanatory variables and potential interactions between them would alter results and to control for potentially confounding and unobserved evolutionary factors (e.g., phylogenetic constraints). The amount of variation explained by taxonomic covariates alone was low, 3% for weeds, and 5% for noxious, whereas in combined models the importance of taxonomic covariates summed to 4.9% for weeds, but 2.1% for noxious (Table 2). While some portion of trait variation in our models is likely to be phylogenetically structured, model performance, based on cross-validation, was only modestly improved by including phylogenetic measures, and trait relationships were not altered.

**Table 2:** Importance value (i.v.) of covariates calculated as the number of times each variable was selected for splitting, weighted by the squared improvement to the model as a result of each split, averaged over all trees, and rescaled to sum to 100 for proportion of weeds and noxious species.

weed					noxious				
trait	i.v.	taxon	i.v.	trend	trait	i.v.	taxon	i.v.	trend
facultative wetland assn.	16.7	Solanales	17.8	+	ln(seed mass)	15.2	Solonales	17.3	+
HCN (s.d.)	14.4	Poaceae	7.8	+	HCN (s.d.)	14.2	Eurosids	13.4	-
ln(seed mass)	11.8	Asteraceae	7.1	+	HCN	13.5	Superrosids	10.7	-
vine	8.0	Eurosids	5.8	-	facultative wetland assn.	10.3	Asteraceae	9.3	+
HCN	7.0	Liliales	4.4	-	ln(max. height)	6.3	Poaceae	8.5	+
annual	6.3	Rosales	4.3	-	annual	5.2	Euphorbiaceae	7.8	+
obligate wetland assn.	5.7	Solanaceae	3.7	-	obligate wetland assn.	4.7	Euasterids	7.4	+
ln(max. height)	4.1	Iridaceae	2.8	-	biennial	4.3	Dipsacales	6.7	+
max. precipitation	3.9	Eudicot	2.7	+	vine	4.1	Rhamnales	5.4	+
subshrub	3.2	Acanthaceae	2.7	-	tree	3.5	Sapindales	5.3	+
perennial	3.2	Monocot-Magnolid	2.6	-	min. precipitation	3.4	Ranunculales	2.4	+
forb	2.9	Poales	2.6	+	min. temperature	3.1	Polypodiales	2.2	-
biennial	2.5	Commelids	2.5	+	perennial	2.7	Solanaceae	2.0	-
min. precipitation	2.4	Asparagales	2.4	-	min. frostfree days	2.6			
tree	2.3	Dipsacales	2.4	+	subshrub	2.6			
min. temperature	2.2	Brassicaceae	2.4	+	forb	2.3			
		Rubiaceae	2.4	-	shrub	2.0			
		Zingiberales	2.1	-					
		Cactaceae	2.1	-					
		Apiaceae	2.1	-					

Covariates with importance values <2% are not included. Trend indicates whether the relationship with taxonomic groups is positive or negative.

Despite the fact that we were able to identify, by contingency analysis, disproportionately invasive genera, we did not find a strong phylogenetic signal in a comparative analysis of invasiveness by genera as a function of biotic traits. However, we did find a weak signal at higher taxonomic levels in the form of a greater propensity for weediness among the Solanales, Poaceae, and Asteraceae, a moderately strong signal of greater propensity for noxiousness among the EuasteridsII, the Solanales, Asteraceae, Poaceae, and Euphorbiaceae, and of a lesser propensity for weediness and noxiousness among the Rosids – among taxonomic groups with large numbers of naturalized species within our study region. The EuasteridsII is made up of the Aquifoliaceae, Araliaceae, Apiaceae, Campanulaceae, Asteraceae, Caprifoliaceae, and Dipsacaceae families. The Rosids includes, among many orders, the Zygophyllales, Celastrales, Oxalidales, Malpighiales, Fabales, Rosales, Fagales, Cucurbitales, Geraniales, Myrtales, Sapindales, Brassicales, and Malvales [18].

Using phylogenetic eigenvectors, recent work on plants of Central European origin [19] found that variance components were greatest for species within genera, much smaller for genera within families, and negligible for higher taxonomic groupings. Our results are not directly comparable, since we were most interested in phylogenetic relationships at the family level and higher, and because phylogenetic effects among closely related species were subsumed by our aggregation at the genus level. Nevertheless, despite some effect at higher levels, the effect of phylogenetic distance in our analyses appears to be primarily at lower (i.e., genera within families) taxonomic levels.

### Characteristics of invasive genera

At the genus level, we attributed 23.6% of propensity for weediness and 28.8% of the propensity for noxiousness to biotic traits. When phylogenetic covariates were included, weed model performance improved to 25.7%, but noxious model performance did not improve. These results suggest that traits conferring a propensity for invasiveness are, in part, a feature of genera. This interpretation is bolstered by considering the strength of this genus-level signal given our aim of generalizing over a broad set of climatic regions and our limitations, e.g.: 1) data were aggregated to genera, but data for some traits were not available for all genera, 2) weed and noxious categories combine many categories of plant pests (e.g. toxic plants, agricultural weeds, natural area and aquatic invaders), and 3) date of introduction and propagule pressure in the form of human cultivation, factors not included in the models for lack of data, are also likely to be important determinants of the currently standing set of invasive species in the U.S. and Canada [20], [21].

**Figure 1:**
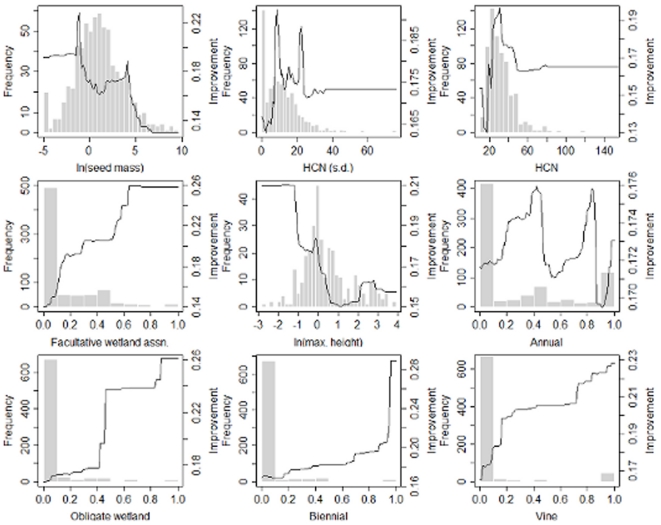
Marginal plots (improvements of the GBM model predicting weed proportion as a function of a single explanatory variable [**46**]) overlaid on a frequency histogram (summarizing over the entire dataset of 760 genera) of each explanatory variable. Right hand y-axis is the log-odds ratio for GBM models. Graphs are arranged by order of explanatory variable importance. HCN =  highest chromosome number mean, and HCN (s.d.)  =  standard deviation in highest chromosome number by genus.

**Figure 2:**
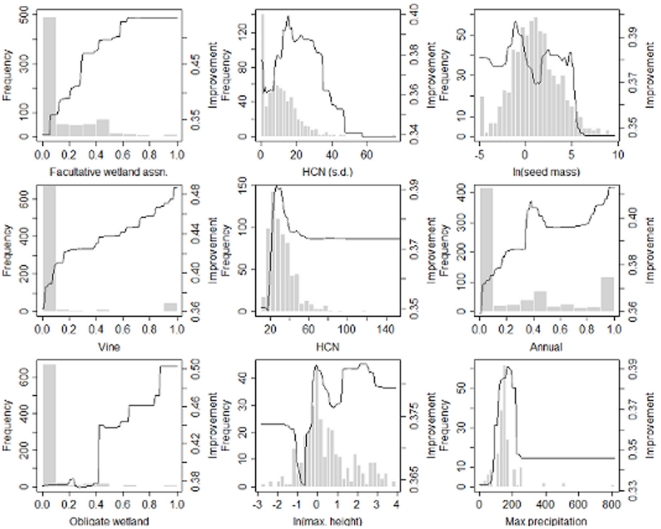
Marginal plots (improvements of the GBM model predicting noxious proportion as a function of a single explanatory variable [**43**]) overlaid on a frequency histogram (summarizing over the entire dataset of 760 genera) of each explanatory variable. Right hand y-axis is the log-odds ratio for final GBM models (using training + validation data sets to train the model). Graphs are arranged by order of explanatory variable importance. HCN =  highest chromosome number mean, and HCN (s.d.)  =  standard deviation in highest chromosome number by genus.

Patterns for both weediness and noxiousness (Table 2) appear to be strongly related to seed mass. Seed mass may serve as a surrogate for dispersal, seedling establishment, and seed production. Prevalence of pests declines sharply in genera when mean seed masses exceeds 1 g. Both weediness and noxiousness also appear to be tied to plasticity in both physiological and life history traits. Genera high in species with facultative wetland habitat affinities are higher in pest species despite the fact that facultative wetland species form a small minority (Figs. 1 and 2). Similarly, genera which are nearly uniformly perennial or annual are least likely to contain noxious species – even though 441 of 760 genera are entirely perennial and 115 entirely annual. Genera with a mix of life histories and/or species which show life history plasticity (multiple labels) are highest in noxious species. The proportion of biennials, a relatively rare group (only 96 of 760 multi-species genera have any), in a genus is also associated with pests of either class. While weediness appears to increase with the mean of maximum height, the proportion of noxious species in genera declines with mean maximum height. Most serious pests fall under 1 m and few exceed 3 m. While several studies have found invasive species to be taller, contrasts were between aliens and natives rather than among aliens [22], [23] or sample sizes were too small to draw strong conclusions [24]. Maximum height might simultaneously encode information on growth form (tall species will typically be woody and/or vines) and somatic growth rate, particularly for annual, biennial, and short-lived perennial species. For predicting noxiousness, prevalence of shrubs and vines are additional factors. Finally, a separate set of pest species are obligately associated with wetland habitats which may, in part, reflect the invasibility of these habitats which experience a high degree of natural and anthropogenic disturbance.

The relationship between chromosome number and prevalence of pests among genera presents a striking pattern. Both weediness and noxiousness are predicted by genus standard deviation for highest chromosome number – variance in chromosome number at the genus level probably reflects the prevalence of polyploid taxa. Interestingly, noxious fraction was positively associated with both highest chromosome number and standard deviation in highest chromosome number, but not with the prevalence of hybrids – suggesting perhaps an association between noxiousness and auto- but not allopolyploids or that hybrids recent enough to be described as hybrids rather than separate species (as in the case of horticultural crosses) are not likely to be invasive (Fig. 2). In addition, genera with intermediate levels of variance in mean number have the highest proportions of weeds, whereas genera with the highest variances have the highest proportions of noxious species (Figs. 1 and 2). These patterns may reflect the importance of polyploidy to successful establishment and spread by non-native plants as has been suggested [25], [26]. Ploidy, hybridization, genome size, and DNA content have all been linked to invasiveness in previous studies [25], [26], [27], [28]. We conjecture that genera with higher means and/or standard deviations for highest chromosome number may contain species that more readily hybridize to form allpolyploids or, in the absence of interspecific hybridization, form autopolyploids.

Summarizing by trait classes, the explanatory variables of weed (a category derived from a Plants National Database watch list)and noxious (a legal class designated by states) fraction show strong similarities in the importance of wetland affinity, seed mass, growth form, life history, and high chromosome number as explanatory variables. Major physiological traits – N-fixation, and photosynthetic pathway – were not explanatory of either weed or noxious fraction. A review of 19 studies of “comparative invasions biology” found the success of plant invaders was generally related to short life cycle, abiotic (mostly wind) dispersal, large native range size, presence of clonal organs, occupation of disturbed habitats, and time since introduction [7]. Although we were able to assess only a subset of these factors in this study, we found that seed mass, a partial surrogate for dispersal, and, to a lesser extent, short (biennial) life-cycles were important explanatory variables in our analyses. Adaptations to wetland habitats may indicate a relationship to disturbance given a relatively high level of both natural and human-induced disturbance associated with many wetland habitats. In addition, facultative wetland associations may serve as a surrogate for an advantageous physiological plasticity. Thus, at the genus level, our results suggest findings additional to those previously reported: an important role for physiological and life history plasticity, and for the ability to generate polyploids in determining the prevalence of species which have invaded successfully enough to be identified as economically damaging.

### Concluding remarks

We have shown that biotic traits are related to invasiveness at the genus level. As a means toward discovering traits which interact to confer a propensity for invasiveness, machine learning methods, which may be used to identify complex non-linear relationships and their interactions, can be an important tool toward this goal. Boosted regression trees explained 24% and 29% of the variation in invasiveness for a large group of naturalized genera in North America, Hawaii, and the Caribbean in terms of biotic traits – a considerable fraction given the that we were not able to control for residence time or propagule pressure, to include species which have been introduced but are not naturalized, and given varying levels of vigilance among states. The two classes of invasive plants considered here – weeds and noxious species – represent human perceptions of economic value and environmental damage. Our results suggest that despite the subjectivity of these classifications, they are underlain by biological differences such as wetland habitat affinities, possibly greater seed production or dispersal relative to competitors, and to advantages perhaps conferred by higher ploidy and/or ability to hybridize. Serious (noxious) pests appear to be more prevalent in genera with higher chromosome numbers and, speculatively, to form polyploid hybrids. Although we identified genera which were disproportionately weedy or noxious, we found relatively little variation in our models that was explained by phylogeny above the genus level. These results indicate that screening at the genus level for traits such as facultative wetland association, mean and standard deviation of highest chromosome number, and seed mass may be an effective first step toward identifying potential invasive plant species.

## Materials and Methods

### Data collection

**Figure 3:**
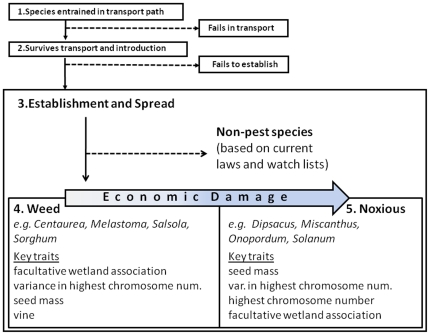
Schematic of the invasion process based on Kolar and Lodge (2001), but emphasizing the divergence in traits corresponding to the two classes of plants, weeds and noxious species.

In the United States and Canada, at least 4,665 non-native species have become naturalized. We compiled a list of species introduced to this region (including Alaska, Hawaii, Puerto Rico, and the Virgin Islands) from the United States Department of Agriculture Plants National Database (http://plants.usda.gov/), which also includes species native and introduced to Canada. Plants National Database defines introduced plants as those that reproduce “spontaneously in the wild without human help”, which we identify with the “establishment” phase in the conceptual framework [10] for the sequence of events constituting a biological invasion (Fig. 3). In this study, we focus on naturalized non-native species and their distributions within the U.S. and Canada.

### Dependent variables

For all genera with more than one introduced species, we tallied the number of introduced species occurring within the study region. Plants National Database lists species as native or introduced (and naturalized) to the lower 48 states, Canada, Alaska, Hawaii, Puerto Rico, and the Virgin Islands. Therefore, for all species listed as naturalized rather than native to a region, we tallied the number of such species per genus. Similarly, for each genus we tallied the number of species designated as either “noxious” by the federal government or at least one state (hereafter “noxious”), or invasive or potentially invasive by “state and federal resource managers, state Exotic Plant Pest Councils, or university noxious weed specialists”. The first class is a legal category, while the second category is a Plants National Database watch list which we refer to generically as “weeds”. These categories are nested subsets such that all noxious species are weeds. From these data, we calculated the fractions of species of each genus that were pests at either level (i.e., number of pests within study region/total number of naturalized alien species in the genus within study region). Of 760 genera (3794 species) with multiple introduced species, noxious species were found in 181 genera, and weeds were found in 348 genera. Hereafter, when we refer to weeds and noxious species collectively, we use the term “invasive species”.

### Explanatory variables

Because we were interested in estimating the relative importance of traits or factors previously found or theorized to be characteristics of invasive plant taxa, we collected data on growth form, life history, seed mass, on breeding system, physiological traits, maximum height, highest chromosome number and hybrid status, and wetland habitat affinity. From Plants National Database, we were able to classify each species according to tree, shrub, sub-shrub, vine, forb, or graminoid, annual, perennial, or biennial, and facultative wetland, obligate wetland, or non-wetland. Because we expected groups of traits listed above to interact with broad physiological traits, we gathered data on photosynthetic pathway [29], [30], and N-fixation [31]. Presence/absence data on apomixis [32] was available at the genus level, and for self-compatibility [33] and dioecy [34] at the family level. Data was complete for the traits above. Growth form, life history and wetland habitat affinity were aggregated as genus means. Individual species can belong to multiple life history classes. Therefore, means capture the number of species per genus which carry each label, annual, perennial, or biennial, and a genus can have the same fraction of species which are annuals as biennials. To assess the effect of climate tolerance on weed and noxious status, we included data from Plants National Database on maximum and minimum precipitation, minimum temperature, and number of frost-free days tolerances. These data were incomplete (data available for only 10% of species), therefore genus means were derived from the means of those species within a genus for which data existed provide n>1. Maximum height data for 1074 species were gathered from Plants National Database, the *Ecological Flora of the British Isles* [35], and the LEDA Traitbase [36], and the *Flora of China*, *Flora of North America*, and *Flora of Pakistan* (available at eFloras.org). We obtained seed mass data from the *Royal Botanic Gardens Kew Seed Information Database* [37] for 2,800 species, and values for highest reported chromosome number for each species from the *Missouri Botanical Gardens Index to Plant Chromosome Numbers* [38] for 2100 species; interspecific hybrids were identified from the Plants National Database. For seed mass, maximum height, and highest chromosome number, we calculated mean values for all genera for which data on more than one species was available. The raw data used for this analysis may be downloaded from the Internet at (*link to be added pending acceptance*).

### Phylogeny

We controlled for phylogeny at levels above genus by using, as additional explanatory variables, an identity matrix derived from published phylogenies [18]. As a proxy for lower level phylogenetic relationships we included the Angiosperm Phylogeny Group II (APG II) family and order designations for each genus. Above the order we employed a speciational model of character evolution by including all nodes (e.g., Monocot, Eudicot, Magnolid) in the APG II supertree setting all branch lengths to be equal. We identified potential phylogenetic non-independence in our analyses by: 1) quantifying improvements in model performance, 2) determining whether the relative importance of explanatory variables shifted with the addition of phylogenetic information, and 3) identifying taxonomic groups which were exceptional. Summarizing, we compiled data for all species, imputed values for missing data, summarized the data as means for all genera containing >1 species, and represented the phylogenetic relationships between genera to produce a data set of genus-level attributes for subsequent analyses.

### Statistical analyses

To test for differences among genera in the proportion of species categorized as weeds, or noxious, we performed serial contingency analyses comparing each genus with all other genera. Pearson χ^2^ contingency tests were performed in R using the coin package [39], with Holm's correction [40] to adjust significance at α = 0.05 for multiple comparisons.

We used machine learning approaches, specifically boosted regression tree analysis, to develop classification models for each class of invasive plants. Machine learning avoids starting with a data model, instead using an algorithm to learn the relationship between response and predictors [41]. Boosted regression trees, which differ from traditional regression methods that produce a single “best” model or tree, rely instead, on boosting, a technique that combines large numbers of relatively simple models adaptively to optimize prediction [41], [42], [43]. Boosted regression trees have important advantages for improving the analysis of large and complex data sets with many independent variables. Like regularized regression, boosted regression trees provide a robust alternative to traditional approaches such as stepwise variable selection. There is no need for prior data transformation or elimination of outliers. Complex nonlinear relationships can be fitted, and interactions between predictors handled automatically. In addition, predictive performance in boosted regression trees is superior to most traditional modeling methods, and despite the complexity of boosted regression tree models (GBMs), they can be summarized to provide mechanistic insights [41], [42]. All results reported here were obtained using the gbm package in R [44] which implements extensions to Friedman's gradient boosting machine [45], [46], [47], and has the additional advantages of handling missing data and of allowing weighting of data.

GBMs were tuned by varying three model constraints: the learning rate, the number of trees, and tree complexity. Model fit was measured with the empirical coefficient of determination, calculated from the model mean squared error and the raw variance
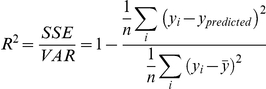
where *y*_predicted_ is generated by the optimized model.

We used cross-validation [42] to determine the optimal learning rate such that the optimum number of trees exceeded 1000. Models were optimized using ten-fold cross-validation. Missing data (missing genus means for explanatory variables) are handled by GBM through surrogate trees [42]. Relative importance of predictor variables was calculated as the number of times each variable was selected for splitting, weighted by the squared improvement to the model as a result of each split, averaged over all trees, and rescaled to sum to 100 [48]. Following the methods above, regression trees were weighted by the number of introduced species per genus to predict the proportion of species in each genus classified as weed or noxious species. Proportion data were arcsin-square root transformed to improve symmetry [49]. Relationships between individual traits and weediness or noxiousness were assessed by marginal plots of the improvement to the GBM model as a function of a single explanatory variable [46].

## Supporting Information

Appendix S1 **Results of contingency analysis of the proportion of species per genus which are weedy or noxious.** Genera in tables are those found disproportionately high in pest species (*p*<0.05).
(DOC)
