# Supplementary material for: Why Are Some Plant Genera More Invasive Than Others?
Source: PLoS One. 2011 Apr 11;6(4):e18654. doi: 10.1371/journal.pone.0018654 (PMC3073987; doi:10.1371/journal.pone.0018654)
Supplement: Appendix S1 — Results of contingency analysis of the proportion of species per genus which are weedy or noxious. Genera in tables are those found disproportionately high in pest species (p<0.05). (DOC) [file pone.0018654.s001.doc]

| **weed genera** | **p-value** | **family** | **order** |
| --- | --- | --- | --- |
| *Carduus* | 0.0000001 | Asteraceae | Asterales |
| *Cuscuta* | 0.0000001 | Cuscutaceae | Solanales |
| *Prosopis* | 0.0000001 | Fabaceae | Fabales |
| *Salsola* | 0.0000001 | Chenopodiaceae | Caryophyllales |
| *Onopordum* | 0.002 | Asteraceae | Asterales |
| *Sorghum* | 0.003 | Poaceae | Cyperales |
| *Melastoma* | 0.005 | Melastomataceae | Myrtales |
| *Centaurea* | 0.006 | Asteraceae | Asterales |
| *Hedychium* | 0.007 | Zingiberaceae | Zingiberales |
| *Tibouchina* | 0.009 | Melastomataceae | Myrtales |
| *Poa* | 0.011 | Poaceae | Cyperales |
| *Avena* | 0.012 | Poaceae | Cyperales |
| *Dipsacus* | 0.012 | Dipsacaceae | Dipsacales |
| *Sonchus* | 0.013 | Asteraceae | Asterales |
| *Tamarix* | 0.014 | Tamaricaceae | Violales |
| *Bromus* | 0.02 | Poaceae | Cyperales |
| *Ligustrum* | 0.022 | Oleaceae | Scrophulariales |
| *Panicum* | 0.023 | Poaceae | Cyperales |
| *Elaeagnus* | 0.027 | Elaeagnaceae | Rhamnales |
| *Ageratina* | 0.031 | Asteraceae | Asterales |
| *Kummerowia* | 0.031 | Fabaceae | Fabales |
| *Bidens* | 0.032 | Asteraceae | Asterales |
| *Triumfetta* | 0.032 | Tiliaceae | Malvales |
| *Ranunculus* | 0.033 | Ranunculaceae | Ranunculales |
| *Imperata* | 0.034 | Poaceae | Cyperales |
| *Prunus* | 0.034 | Rosaceae | Rosales |
| *Schismus* | 0.034 | Poaceae | Cyperales |
| *Cosmos* | 0.036 | Asteraceae | Asterales |
| *Melochia* | 0.037 | Sterculiaceae | Malvales |
| *Tribulus* | 0.037 | Zygophyllaceae | Sapindales |
| *Xanthium* | 0.037 | Asteraceae | Asterales |
| *Limnophila* | 0.038 | Scrophulariaceae | Scrophulariales |
| *Coronopus* | 0.039 | Brassicaceae | Capparales |
| *Macaranga* | 0.039 | Euphorbiaceae | Euphorbiales |
| *Oryza* | 0.039 | Poaceae | Cyperales |
| *Paederia* | 0.039 | Rubiaceae | Rubiales |
| *Pueraria* | 0.039 | Fabaceae | Fabales |
| *Ipomoea* | 0.04 | Convolvulaceae | Solanales |
| *Emex* | 0.041 | Polygonaceae | Polygonales |
| *Myriophyllum* | 0.041 | Haloragaceae | Haloragales |
| *Raphanus* | 0.042 | Brassicaceae | Capparales |
| *Psidium* | 0.044 | Myrtaceae | Myrtales |
| *Cortaderia* | 0.045 | Poaceae | Cyperales |
| *Brassica* | 0.046 | Brassicaceae | Capparales |
| *Lygodium* | 0.046 | Lygodiaceae | Polypodiales |
| *Salix* | 0.046 | Salicaceae | Salicales |
| *Citharexylum* | 0.047 | Verbenaceae | Lamiales |
| *Eichhornia* | 0.048 | Pontederiaceae | Liliales |

| **noxious genera** | **p-value** | **family** | **order** |
| --- | --- | --- | --- |
| *Carduus* | 0.0000001 | Asteraceae | Asterales |
| *Centaurea* | 0.0000001 | Asteraceae | Asterales |
| *Dipsacus* | 0.0000001 | Dipsacaceae | Dipsacales |
| *Miscanthus* | 0.0000001 | Poaceae | Cyperales |
| *Onopordum* | 0.0000001 | Asteraceae | Asterales |
| *Prosopis* | 0.0000001 | Fabaceae | Fabales |
| *Salsola* | 0.0000001 | Chenopodiaceae | Caryophyllales |
| *Solanum* | 0.0000001 | Solanaceae | Solanales |
| *Sorghum* | 0.002 | Poaceae | Cyperales |
| *Oryza* | 0.006 | Poaceae | Cyperales |
| *Pueraria* | 0.006 | Fabaceae | Fabales |
| *Emex* | 0.007 | Polygonaceae | Polygonales |
| *Lonicera* | 0.008 | Caprifoliaceae | Dipsacales |
| *Triumfetta* | 0.008 | Tiliaceae | Malvales |
| *Imperata* | 0.009 | Poaceae | Cyperales |
| *Paederia* | 0.009 | Rubiaceae | Rubiales |
| *Lygodium* | 0.01 | Lygodiaceae | Polypodiales |
| *Myriophyllum* | 0.01 | Haloragaceae | Haloragales |
| *Pennisetum* | 0.01 | Poaceae | Cyperales |
| *Eichhornia* | 0.011 | Pontederiaceae | Liliales |
| *Hieracium* | 0.013 | Asteraceae | Asterales |
| *Limnophila* | 0.014 | Scrophulariaceae | Scrophulariales |
| *Euphorbia* | 0.019 | Euphorbiaceae | Euphorbiales |
| *Hedera* | 0.022 | Araliaceae | Apiales |
| *Cardaria* | 0.024 | Brassicaceae | Capparales |
| *Cynanchum* | 0.025 | Asclepiadaceae | Gentianales |
| *Senecio* | 0.026 | Asteraceae | Asterales |
| *Orobanche* | 0.027 | Orobanchaceae | Scrophulariales |
| *Rorippa* | 0.035 | Brassicaceae | Capparales |
| *Spartina* | 0.035 | Poaceae | Cyperales |
| *Carthamus* | 0.038 | Asteraceae | Asterales |
| *Cuscuta* | 0.038 | Cuscutaceae | Solanales |
| *Hyptis* | 0.039 | Lamiaceae | Lamiales |
| *Ligustrum* | 0.039 | Oleaceae | Scrophulariales |
| *Tamarix* | 0.039 | Tamaricaceae | Violales |
| *Elaeagnus* | 0.049 | Elaeagnaceae | Rhamnales |
